# The Influence of the CB1 Receptor Ligands on the Schizophrenia-Like Effects in Mice Induced by MK-801

**DOI:** 10.1007/s12640-016-9662-0

**Published:** 2016-08-30

**Authors:** Marta Kruk-Slomka, Barbara Budzynska, Tomasz Slomka, Izabela Banaszkiewicz, Grazyna Biala

**Affiliations:** 1 Department of Pharmacology and Pharmacodynamics, Medical University of Lublin, Chodzki 4a Street, 20-093 Lublin, Poland; 2 Department of Mathematics and Medical Biostatistics, Medical University of Lublin, Jaczewskiego 4 Street, 20-954 Lublin, Poland

**Keywords:** Schizophrenia, Endocannabinoid system, CB1 receptor ligands, NMDA receptor antagonist, Mice

## Abstract

A growing body of psychiatric research has emerged, focusing on the role of endocannabinoid system in psychiatric disorders. For example, the endocannabinoid system, via cannabinoid CB (CB1 and CB2) receptors, is able to control the function of many receptors, such as *N*-methyl-d-aspartate (NMDA) receptors connected strictly with psychosis or other schizophrenia-associated symptoms. The aim of the present research was to investigate the impact of the CB1 receptor ligands on the symptoms typical for schizophrenia. We provoked psychosis-like effects in mice by an acute administration of NMDA receptor antagonist, MK-801 (0.1–0.6 mg/kg). An acute administration of MK-801 induced psychotic symptoms, manifested in the increase in locomotor activity (hyperactivity), measured in actimeters, as well as the memory impairment, assessed in the passive avoidance task. We revealed that an acute injection of CB1 receptor agonist, oleamide (5–20 mg/kg), had no influence on the short- and long-term memory-related disturbances, as well as on the hyperlocomotion in mice, provoking by an acute MK-801. In turn, an amnestic effects or hyperactivity induced by an acute MK-801 was attenuated by an acute administration of AM 251 (0.25–3 mg/kg), a CB1 receptor antagonist. The present findings confirm that endocannabinoid system is able to modify a variety of schizophrenia-like responses, including the cognitive disturbances and hyperlocomotion in mice. Antipsychotic-like effects induced by CB1 receptor antagonist, obtained in our research, confirm the potential effect of CB1 receptor blockade and could have important therapeutic implications on clinical settings, in the future.

## Introduction

Schizophrenia is a chronic, severe, mental disorder usually characterized by abnormal social behavior. The main symptoms of schizophrenia are often grouped into three major diagnostic classes: positive, negative (or deficit) symptoms, and cognitive disorders (Lewis and Liberman 2000).

It has been known that the function of different neurotransmitters systems, such as dopaminergic system, glutamatergic system, gamma-aminobutyric (GABA)-related system, or/and endocannabinoid system, is altered in psychosis (Broome et al. 2005; Carlsson 2004). The most known hypothesis for schizophrenia is the glutamate (Glu)-related hypothesis. Glu is the major excitatory neurotransmitter in the brain which has a leading role in neural physiology, especially in mechanisms of synaptic plasticity, such as the long-term potentiation (LTP) and long-term depression underlying cellular basis of some phases of memory and learning (Riedel et al. 2003; Shapiro 2001). It has been revealed that glutamatergic transmission through *N*-methyl-d-aspartate (NMDA)-type receptors is strictly implicated in specific symptoms of schizophrenia, such as psychosis. Furthermore, glutamatergic hypofunction, closely associated with NMDA receptors hypofunction, is currently believed to provoke dopaminergic deregulation observed in the brain of patients with schizophrenia (Harrison and Weinberger 2005; Javitt 2007) and underlie the symptoms recognized as schizophrenia (Mohn et al. 1999; Stone et al. 2007). Therefore, agonists of NMDA receptors may have the potential to attenuate the symptoms of schizophrenia, while antagonists of these receptors produce psychotic symptoms and others schizophrenia-associated symptoms (Abi-Saab et al. 1998).

A variety of pre-clinical and clinical studies have indicated that endocannabinoid system participate in many central pathways connected with psychosis-like state, including glutamatergic transmission. In hippocampal slice cultures (Khaspekov et al. 2004), and in cultured neurons (Kim et al. 2006), it has been demonstrated that endocannabinoid system through cannabinoid (CB: CB1 and CB2) receptors is involved in the control of NMDA receptors-related neuronal dysregulation, connected with schizophrenia-like symptoms. This relationship has been confirmed in behavioral studies (Liu et al. 2009; Marsicano et al. 2003). For example, in animal models, it has been demonstrated that CB1 receptor agonists often induced cognitive impairments in rodents (Ferrari et al. 1999; Kruk-Slomka and Biala 2016; Pamplona and Takahashi 2006), whereas the antagonism of CB1 receptors generally enhanced rodent performance in variety memory tasks (Kruk-Slomka and Biala 2016; Lichtman 2000; Takahashi et al. 2005; Terranova et al. 1996). Additionally, CB1 receptor agonists induced psychosis-like symptoms, and CB1 receptor antagonists had antipsychotic properties evaluated in animal models of schizophrenia (Almeida et al. 2014; Levin et al. 2012; Roser and Haussleiter 2012).

Although the biochemical, molecular, and pharmacological studies demonstrating functional interactions between the glutamatergic and endocannabinoid system are available (Rodríguez-Muñoz et al. 2012; Sánchez-Blázquez et al. 2014), many other interactions in the context of schizophrenia have not been yet examined. Therefore, the aim of presented studies was to evaluate the psychotic potential of CB1 receptor ligands and their influence on the psychosis-related symptoms in mice. Our experiments have primarily focused on the complex implication of the CB1 receptor subtype in the schizophrenia-associated symptoms in mice, using a pharmacological animal model of schizophrenia. We used a potent agonist of CB1 receptor, oleamide, and a selective CB1 receptor antagonist, AM 251. To provoke the symptoms of schizophrenia in mice, we used NMDA receptor antagonist, MK-801. It has been known that an acute inhibition of NMDA receptors, e.g., by MK-801, provokes schizophrenia-like behavior in mice, both positive and negative symptoms of this disorder, and shows phenomenological validity as a model. This model is often used to predict the effect of many compounds with potential antipsychotic properties (Large 2007). Concerning animal models, it is well assumed that an acute administration of MK-801 in rodents induces psychotic symptoms, manifested in the increase in locomotor activity (hyperactivity) and memory impairment. Hyperlocomotion in rodents has been correlated with the positive clinical symptoms of schizophrenia (psychosis); in turn, memory-related disturbances evaluated in animal tests have been correlated to the cognitive deficits in humans (Arnt and Skarsfeldt 1998; Bubenίkova-Valesova et al. 2008; Micale et al. 2013; Peleg-Raibstein et al. 2012). In our studies, a different stages (acquisition, consolidation and retrieval) of short- and long-term memory-related responses in mice were measured in the commonly used animal model of memory—the passive avoidance (PA) task—while locomotion was measured in actimeters.

The results of this study will help increase knowledge on the role of CB1 receptors, in the positive as well as cognitive symptoms typical for schizophrenia, including interactions between these receptors with other receptors strictly associated with schizophrenia, e.g., NMDA receptors. Perhaps, our results can be used to initiate new research to clinical level and more effective strategies for the control/attenuation of symptoms of schizophrenia and/or other similar psychotic disorders.

## Materials and Methods

### Animals

The experiments were carried out on naive male Swiss mice (Farm of Laboratory Animals, Warszawa, Poland) weighing 20–30 g. The animals were maintained under standard laboratory conditions (12-h light/dark cycle, room temperature 21 ± 1 °C) with free access to tap water and laboratory feed (Agropol, Motycz, Poland) in their home cages, and adapted to the laboratory conditions for at least 1 week. Each experimental group consisted of 8–12 animals. All behavioral experiments were performed between 8:00 and 15:00, and were conducted according to the National Institute of Health Guidelines for the Care and Use of Laboratory Animals and to the European Community Council Directive for the Care and Use of laboratory animals of 22 September 2010 (2010/63/EU) and approved by the local ethics committee.

### Drugs

The tested compounds were as follows:

- Oleamide (5, 10, 20 mg/kg) (Tocris, USA)—CB1 receptor agonist.
- AM 251 (0.25, 0.5, 1, 3 mg/kg) (Tocris, USA)—CB1 receptor antagonist.
- MK-801 (0.1, 0.3, 0.6 mg/kg) (Tocris, USA)—NMDA receptor antagonist.

All CB compounds and MK-801 were suspended in a 1 % solution of Tween 80 (Sigma, St. Louis, MO, USA) in saline solution (0.9 % NACl) and administered intraperitoneally (ip) at a volume of 10 ml/kg. Fresh drug solutions were prepared on each day of experimentation. Control groups received injections of saline with Tween 80 (vehicle) at the same volume and by the same route of administration.

Experimental doses of drugs used and procedures were selected on the basis of literature data (Akanmu et al. 2007; Barzegar et al. 2015; Bialuk and Winnicka 2011; Bubeníková-Valesová et al. 2010; Javadi-Paydar et al. 2012; Murillo-Rodríguez et al. 2001; Nestler and Hyman 2010), our previous experiments (Biala and Kruk 2008; Biala et al. 2009; Budzynska et al. 2009; Kruk-Slomka et al. 2015a, b; Kruk-Slomka and Biala 2016), and preliminary studies.

### Experimental Procedures

We used an animal model of schizophrenia. The used procedure is commonly accepted (Bubeníková-Valesová et al. 2010; Nestler and Hyman 2010) and is based on the psychotic properties of NMDA receptor antagonist, e.g., MK-801. We provoked the schizophrenia-like behaviors (cognitive disturbances and hyperlocomotion) in mice by an acute administration of MK-801.

Next, we evaluated the influence of CB1 receptor ligands, oleamide and AM 251, on the above schizophrenia-like effects in mice provoked by MK-801. Memory-related responses in mice were measured in the PA task, locomotion was measured in actimeters.

In the presented experiments, we used an independent groups of mice for each kind of behavioral experiment (separate group of mice for the assessment of memory-related effects and separate group of mice for the assessment of locomotor activity), for each drug and dose.

### Memory-Related Responses

The apparatus of the PA consisted of two-compartment acrylic box with a lighted compartment (10 × 13 × 15 cm) and darkened compartment (25 × 20 × 15 cm). The light chamber was illuminated by a fluorescent light (8 W) and was connected to the dark chamber which was equipped with an electric grid floor. Entrance of animals to the dark box was punished by an electric foot shock (0.2 mA for 2 s).

On the first day of training (pretest), mice were placed individually into the light compartment and allowed to explore the light box. After 30 s, the guillotine door was raised to allow the mice to enter the dark compartment. When the mice entered to the dark compartment, the guillotine door was closed and an electric foot shock (0.2 mA) of 2 s duration was delivered immediately to the animal via grid floor. The latency time for entering the dark compartment was recorded (TL1). The mouse which did not enter spontaneously into the dark box within 300 s was excluded from further tests. In the subsequent trial (retention), the same mice were again placed individually in the light compartment of the PA apparatus. After a 30-s adaptation period in the light (safe) chamber, the door between the compartments was raised and the time taken to re-enter the dark compartment was recorded (TL2). No foot shock was applied in this trial. Basically, in this kind of procedure, when the mouse did not enter spontaneously into the dark box within 300 s, the test was stopped.

Depending on the procedure used, PA test allows examining different durations of memory (short-term and long-term memory) according to the period between training and test, as well as different stages of memory (acquisition, consolidation, and retrieval) according to the time of drug treatment.

When mice were tested 2 h after TL1, the short-term memory was assessed, whereas longer time (24 h) allows assess long-term memory processes. Drug administration before the first trial (before pretest) should interfere with the acquisition of information, and drug administration immediately after the first trial (after pretest) should exert an effect on the process of consolidation, while the administration of tested compounds before the second trial (before test) should interfere with the retrieval of memory information (Allami et al. 2011; Javadi-Paydar et al. 2012; Kruk-Slomka et al. 2015a; Kruk-Slomka and Biala 2016).

### Locomotion

Locomotion of mice was recorded individually in round actimeter cages (Multiserv, Lublin, Poland; 32 cm in diameter, two light beams) kept in a sound-attenuated experimental room. Two photocell beams, located across the axis, automatically measured animal’s movements. The horizontal locomotor activity, i.e., the number of photocell beam breaks, was automatically measured with a 20-min interval for 200 min (Mohn et al. 1999; Zhou et al. 2012).

### Treatment

#### For Memory-Related Responses

The first step of experiment was designed to estimate the influence of MK-801 (0.1, 0.3 and 0.6 mg/kg; ip) on the different stages of short- as well as long-term memory in mice, using the PA test.

For the *acquisition of memory*, MK-801 or vehicle, for the control group, was administered 30 min before the first trial, and mice were re-tested after 2 h (short-term memory) or after 24 h (long-term memory). For the *consolidation of memory*, MK-801 or vehicle, for the control group, was injected immediately after the first trial, and mice were re-tested after 2 h or after 24 h. Finally, for the *retrieval of memory*, MK-801 or vehicle, for the control group, was injected 30 min before retrieval and that retrieval was carried out 2 or 24 h after the first trial (Table 1).

**Table 1:** The scheme of MK-801 or vehicle administration during the assessment of short- and long-term memory acquisition (A), consolidation (B), or retrieval (C) in the PA test

A. Acquisition of memory					
	Drug administration	Interval	TL1	Interval	TL2
Short-term memory	MK-801 (0.1–0.6 mg/kg) or vehicle	30 min	+	2 h	+
Long-term memory	MK-801 (0.1–0.6 mg/kg) or vehicle	30 min	+	24 h	+

B. Consolidation of memory					
	TL1	Interval	Dug administration	Interval	TL2
Short-term memory	+	0 min	MK-801 (0.1–0.6 mg/kg) or vehicle	2 h	+
Long-term memory	+	0 min	MK-801 (0.1–0.6 mg/kg) or vehicle	24 h	+

C. Retrieval of memory					
	TL1	Interval	Drug administration	Interval	TL2
Short-term memory	+	2 h	MK-801 (0.1–0.6 mg/kg) or vehicle	30 min	+
Long-term memory	+	24 h	MK-801 (0.1–0.6 mg/kg) or vehicle	30 min	+

Based on this pilot experiment, we have chosen the most effective doses of MK-801 in the PA test in mice for the next experiments with CB1 receptor ligands. After that, based on the available literature data (Barzegar et al. 2015) and primarily on the results obtained from our previous experiments (Kruk-Slomka and Biala 2016), in which we determined the effects of an acute injection of different doses of oleamide (5–20 mg/kg), a CB1 receptor agonist and different doses of AM 251 (0.25–3 mg/kg), a CB1 receptor antagonist, on the short-term or long-term memory stages in the inhibitory avoidance (IA) task in mice, we have chosen the non-effective dose of oleamide (5 mg/kg) and AM 251 (0.25 mg/kg) for the next experiment with MK-801. We evaluated the influence of oleamide and AM 251 on the memory-related responses induced by MK-801 in the PA task.

Non-effective oleamide (5 mg/kg; ip) (Kruk-Slomka and Biala 2016) or vehicle was administered acutely 15 min before an acute injection of MK-801 (0.3 mg/kg, ip) or vehicle. Similarly, non-effective dose of AM 251 (0.25 mg/kg, ip) (Kruk-Slomka and Biala 2016) or vehicle were administered acutely 15 min before an acute injection of MK-801 (0.3 mg/kg, ip) or vehicle. The mice were then tested for *acquisition, consolidation,* and *retrieval* of short- and long-term memory in the same scheme described above and presented in the Table 2.

**Table 2:** The scheme of oleamide (5 mg/kg) or AM 251 (0.25 mg/kg) and MK-801 (0.3 mg/kg) co-administration during the assessment of short- and long-term memory acquisition (A), consolidation (B), or retrieval (C) in the PA test

A. Acquisition of memory							
	Drug administration	Interval	Drug administration	Interval	TL1	Interval	TL2
Short-term memory	oleamide (5 mg/kg) or AM (0.25 mg/kg) or vehicle	15 min	MK-801 (0.3 mg/kg) or vehicle	15 min	+	2 h	+
Long-term memory	oleamide (5 mg/kg) or AM (0.25 mg/kg) or vehicle	15 min	MK-801 (0.3 mg/kg) or vehicle	15 min	+	24 h	+

B. Consolidation of memory							
	TL1	Interval	Drug administration	Interval	Drug administration	Interval	TL2
Short-term memory	+	0 min	oleamide (5 mg/kg) or AM (0.25 mg/kg) or vehicle	15 min	MK-801 (0.3 mg/kg) or vehicle	2 h	+
Long-term memory	+	0 min	oleamide (5 mg/kg) or AM (0.25 mg/kg) or vehicle	15 min	MK-801 (0.3 mg/kg) or vehicle	24 h	+

C. Retrieval of memory							
	TL1	Interval	Drug co-administration	Interval	Drug administration	Interval	TL2
Short-term memory	+	2 h	oleamide (5 mg/kg) or AM (0.25 mg/kg) or vehicle	15 min	MK-801 (0.3 mg/kg) or vehicle	15 min	+
Long-term memory	+	24 h	oleamide (5 mg/kg) or AM (0.25 mg/kg) or vehicle	15 min	MK-801 (0.3 mg/kg) or vehicle	15 min	+

#### For Psychotic-Like Symptoms

Horizontal locomotor activity was measured immediately after an acute injection of MK-801 (0.1; 0.3; 0.6 mg/kg; ip), oleamide (5; 10; 20 mg/kg, ip), AM 251 (0.25; 0.5; 1 and 3 mg/kg, ip), or vehicle for the control group. Next, we evaluated the impact of an acute administration of oleamide (5–20 mg/kg, ip) or AM 251 (0.25–3 mg/kg, ip) on the hyperlocomotion of mice provoked by an acute MK-801 (0.1–0.6 mg/kg, ip). For this purpose, oleamide, AM 251, or vehicle were administered 15 min before injection of MK-801 or vehicle. The mice were then tested immediately after the last injection (Table 3).

**Table 3:** The scheme of drugs (MK-801, oleamide, AM 251) or vehicle administration (A) and drugs co-administration (B) during the assessment of locomotor activity of mice

A. Locomotor activity		
Drug administration	Interval	Measurement of locomotor activity for 200 min
MK-801 (0.1–0.6 mg/kg), oleamide (5–20 mg/kg), AM 251 (0.25–3 mg/kg) or vehicle	0 min	+

B. Locomotor activity				
Drugs administration	Interval	Drug administration	Interval	Measurement of locomotor activity for 200 min
oleamide (5 mg/kg) or AM 251 (0.25 and 0.5 mg/kg) or vehicle	15 min	MK-801 (0.3 and 0.6 mg/kg) or vehicle	0 min	+

### Statistical Analysis

The statistical analysis was performed using one-way analysis of variance (ANOVA) or two-way ANOVA—for the factors of pretreatment (oleamide or AM 251), treatment (MK 801), and pretreatment/treatment interactions for the memory-related responses or for the factors of time, drugs, and time/drugs interactions for the psychotic-like symptoms.

Post hoc comparison of means was carried out with the Tukey’s test (for one-way ANOVA) or with the Bonferroni’s test (for two-way ANOVA) for multiple comparisons, when appropriate. The data were considered statistically significant at confidence limit of *p* < 0.05. ANOVA analysis with Tukey’s or Bonferroni’s post-tests was performed using GraphPad Prism version 5.00 for Windows, GraphPad Software, San Diego California USA, www.graphpad.com.

For the memory-related responses, the changes in PA performance were expressed as the difference between retention and training latencies and were taken as a latency index (LI). LI was calculated for each animal and reports as the ratio: LI = TL2−TL1/TL1, where *TL1* is the time taken to enter the dark compartment during the training and *TL2* is the time taken to re-enter the dark compartment during the retention (Chimakurthy and Talasila 2010).

For the psychotic-like symptoms, the horizontal locomotor activity, i.e., the number of photocell beam breaks, was measured.

## Results

First, we induced the memory disturbances characteristic for schizophrenia (negative symptoms), by the acute administration of MK-801, and evaluated the influence of CB1 receptor ligands on these memory impairment provoked by MK-801.

### Memory-Related Disturbances in the PA Test in Mice Provoked by an Acute Administration of MK-801

#### Acquisition of Memory

**Fig. 1:**
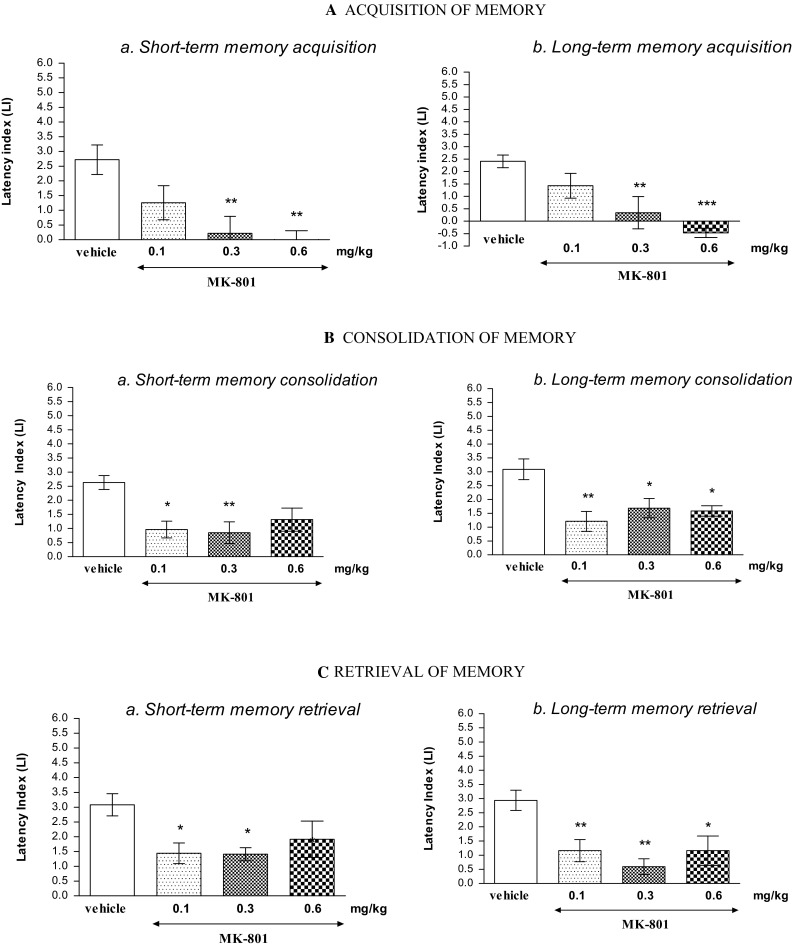
Effects of an acute MK-801 or saline administration on the latency index (LI) during the short-term or long-term acquisition trial **(A),** consolidation trial **(B),** and retrieval trial **(C)**, using the PA test in mice. MK-801 (0.1; 0.3 and 0.6 mg/kg; ip) or vehicle was injected 30 min before the first trial (**A**) or immediately after first trial (**B**), and mice were re-tested 2 h [for short-term memory (**a**)] or 24 h [for long-term memory (**b**)] later. In the case of retrieval of memory (**C**), oleamide, MK-801 (0.1, 0.3 and 0.6 mg/kg; ip) or vehicle was administered 2 h (*a*) or 24 h (*b*) after the first trial,and mice were re-tested 30 min after the last injection; *n* = 8–12; the mean ± SEM; *p *<* 0.05; **p *<* 0.01; ***p *<* 0.001 vs. vehicle-treated control group; Tukey’s test

One-way ANOVA revealed that administration of acute ip doses of MK-801 (0.1; 0.3 and 0.6 mg/kg) had a statistically significant effect on LI values for short-term memory acquisition [*F*(3.31) = 6.283; *p* = 0.0021], as well as for long-term memory acquisition [*F*(3.32) = 8.619; *p* = 0.0003]. Indeed, the post hoc Tukey’s test confirmed that the treatment with MK-801 (0.3 and 0.6 mg/kg) significantly decreased LI values in mice compared to those in the vehicle-treated control group (*p* < 0.01—for short-term memory acquisition (Fig. 1Aa), and *p* < 0.01; *p* < 0.001—for long-term memory acquisition, for the dose of 0.3 and 0.6 mg/kg, respectively) (Fig. 1Ab), indicating that MK-801, at these used doses, impaired both the short- and long-term acquisition of memory and learning.

####  Consolidation of Memory

One-way ANOVA indicated that administration of acute ip doses of MK-801 (0.1; 0.3 and 0.6 mg/kg) had a statistically significant effect on LI values for short-term memory consolidation [*F*(3.32) = 5.585; *p* = 0.0038], as well as for long-term memory consolidation [*F*(3.29) = 6.436; *p* = 0.0021]. Indeed, treatment with MK-801 significantly decreased LI values in mice compared to those in the vehicle-treated control group for short-term memory consolidation (*p* < 0.05 for dose of 0.1 mg/kg; *p* < 0.01 for dose of 0.3 mg/kg; Tukey’s test) (Fig. 1Ba), and for long-term memory consolidation (*p* < 0.05 for dose of 0.3 mg/kg and 0.6 mg/kg; *p* < 0.01 for dose of 0.1 mg/kg; Tukey’s test) (Fig. 1Bb), indicating that MK-801, at these used doses, impaired the short- and/or long-term consolidation of memory and learning.

#### Retrieval of Memory

One-way ANOVA indicated that administration of acute ip doses of MK-801 (0.1; 0.3 and 0.6 mg/kg) had a statistically significant effect on LI values for short-term memory retrieval [*F*(3.28) = 3.777; *p* = 0.0231], as well as for long-term memory consolidation [*F*(3.32) = 7.284; *p* = 0.0009]. Indeed, treatment with MK-801 significantly decreased LI values in mice compared to those in the vehicle-treated control group for short-term memory retrieval (*p* < 0.05 for dose of 0.1 and 0.3 mg/kg; Tukey’s test) (Fig. 1Ca), and for long-term memory retrieval (*p* < 0.05 for dose of 0.6 mg/kg; *p* < 0.01 for dose of 0.1 and 0.3 mg/kg; Tukey’s test) (Fig. 1Cb), indicating that MK-801, at these used doses, impaired the short- and/or long-term retrieval of memory and learning.

In our previously published experiments, we revealed that an acute injection of oleamide (10 and 20 mg/kg), a CB1 receptor agonist, diminished the short-term as well as long-term acquisition, consolidation/retention, and/or retrieval of memory and learning in the IA task. In turn, an acute injection of AM 251 (1 and 3 mg/kg), a CB1 receptor antagonist, improved stages of the short-term or long-term memory, mentioned above. This memory impairment induced by effective dose of oleamide (20 mg/kg) was reversed by a non-effective dose of CB1 receptor antagonist, AM 251 (0.25 mg/kg) in mice using the IA test, confirming that the CB1 receptor-related mechanism is one of the possible mechanisms responsible for memory and learning responses (Kruk-Slomka and Biala 2016).

Therefore, based on the results obtained from these cited experiments (Kruk-Slomka and Biala 2016), the non-effective dose of oleamide (5 mg/kg) and non-effective dose of AM 251 (0.25 mg/kg) were then chosen for the next behavioral experiment evaluating the influence of these CB1 receptor ligands on the above-described memory impairment, provoked by an acute injection of MK-801 (0.3 mg/kg), using the PA test in mice.

### The Influence of the Administration of Oleamide on the Memory Impairment Provoked by an Acute Administration of MK-801 in the PA Test in Mice

#### Acquisition of Memory

**Fig. 2:**
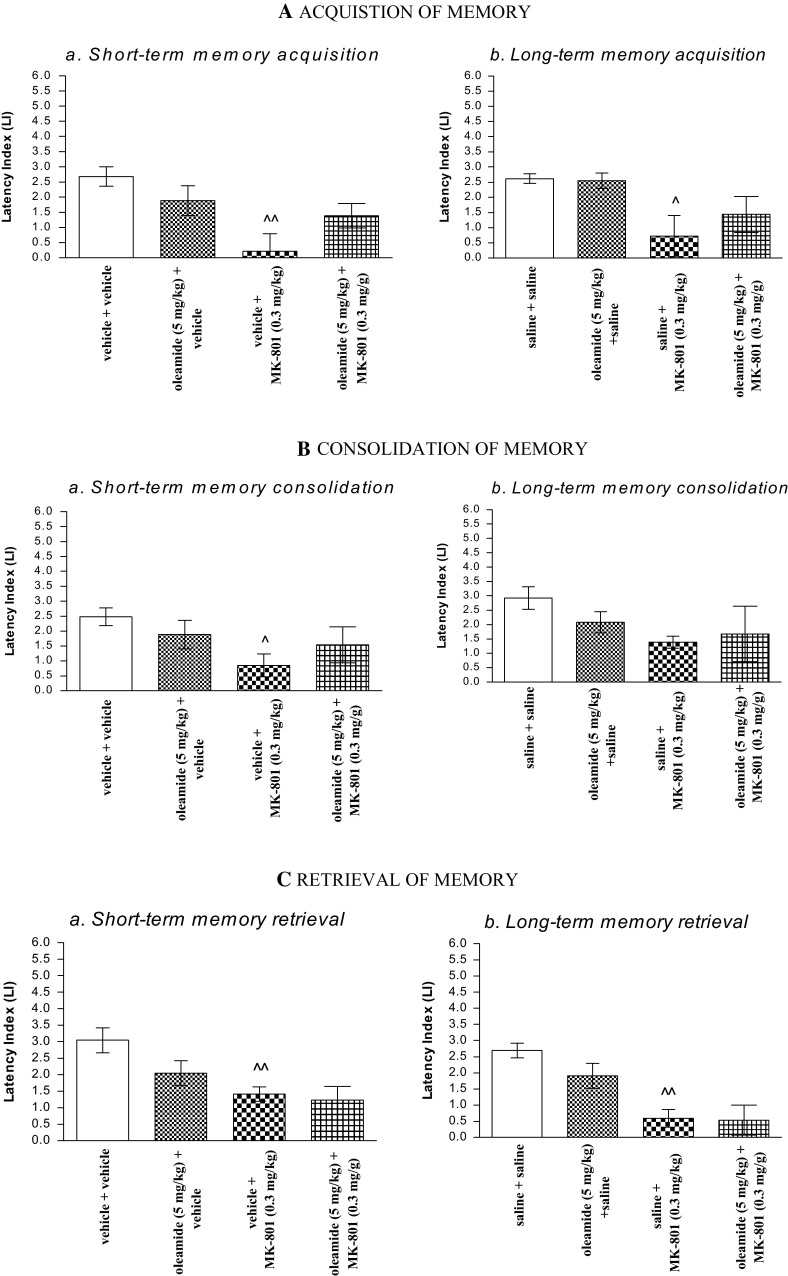
Influence of oleamide on the memory-related responses, expressed as latency index (LI) during the short-term (**a**) or long-term (**b**) acquisition **(A),** consolidation **(B**), and retrieval **(C)** trial, induced by an acute administration of MK-801, using the PA test in mice. Non-effective dose of oleamide (5 mg/kg, ip) or vehicle was administered 15 min prior to vehicle or effective (0.3 mg/kg, ip) MK-801 injection. All drugs were administered 15 min before the first trial (**A**) or immediately after the first trial (**B**), and mice were re-tested 2 h (for short-term memory) or 24 h (for long-term memory) later. In the case of retrieval of memory (**C**), all drugs were administered 2 h (**a**) or 24 h (**b**) after the first trial, and mice were re-tested 15 min after the last injection; *n* = 8–12; the mean ± SEM; ^*p* *<* 0.05; ^^*p* *<* 0.01 vs. vehicle/vehicle-treated group; two-way ANOVA/Bonferroni test

For short-term memory acquisition, two-way ANOVA analyses revealed that there was no statistically significant effect caused by oleamide (5 mg/kg) pretreatment [*F*(1.28) = 0.18; *p* = 0.6766], but there was a statistically significant effect caused by MK-801 (0.3 mg/kg) treatment [*F*(1.28) = 11.40; *p* = 0.0022] and interactions [*F*(1.28) = 5.02; *p* = 0.0331]. The post hoc Bonferroni’s test confirmed that MK-801 at the dose of 0.3 mg/kg significantly decreased LI values in mice in the PA test in comparison to the vehicle/vehicle-treated mice, pointing to the amnestic effect of this drug (*p* < 0.01). However, oleamide (5 mg/kg) had no influence on this amnestic effect of MK-801 (0.3 mg/kg) (Fig. 2Aa).

For long-term memory acquisition, two-way ANOVA analyses revealed that there was no statistically significant effect caused by oleamide (5 mg/kg) pretreatment [*F*(1.32) = 0.46; *p* = 0.5003] as well as by interactions between oleamide (5 mg/kg) pretreatment and MK-801 (0.3 mg/kg) treatment [*F*(1.32) = 0.68; *p* = 0.4170], but there was a statistically significant effect caused by MK-801 (0.3 mg/kg) treatment [*F*(1.32) = 9.97; *p* = 0.0035]. The post hoc Bonferroni’s test revealed that MK-801 at the dose of 0.3 mg/kg significantly decreased LI values in mice in the PA test in comparison to the vehicle/vehicle-treated mice, confirming the amnestic effect of this drug (*p* < 0.05). Oleamide (5 mg/kg) had no influence on this amnestic effect of MK-801 (0.3 mg/kg) (Fig. 2Ab).

#### Consolidation of Memory

For short-term memory consolidation, two-way ANOVA analyses revealed that there was no statistically significant effect caused by oleamide (5 mg/kg) pretreatment [*F*(1.28) = 0.01; *p* = 0.9200] as well as by interactions between oleamide (5 mg/kg) pretreatment and MK-801 (0.3 mg/kg) treatment [*F*(1.28) = 2.20; *p* = 0.1496], but there was a statistically significant effect caused by MK-801 (0.3 mg/kg) treatment [*F*(1.28) = 5.11; *p* = 0.0318]. The post hoc Bonferroni’s test revealed that MK-801 at the dose of 0.3 mg/kg significantly decreased LI values in mice in the PA test in comparison to the vehicle/vehicle-treated mice, confirming the amnestic effect of this drug (*p* < 0.05). Oleamide (5 mg/kg) had no influence on this amnestic effect of MK-801 (0.3 mg/kg) (Fig. 2Ba).

For long-term memory consolidation, two-way ANOVA analyses revealed that there was no statistically significant effect caused by oleamide (5 mg/kg) pretreatment [*F*(1.25) = 0.20; *p* = 0.6585], MK-801 (0.3 mg/kg) treatment [*F*(1.25) = 2.49; *p* = 0.1270], as well as by interactions between oleamide (5 mg/kg) pretreatment and MK-801 (0.3 mg/kg) treatment [*F*(1.25) = 0.84; *p* = 0.3694]. Oleamide (5 mg/kg) had no influence on this amnestic effect of MK-801 (0.3 mg/kg) (Fig. 2Bb).

#### Retrieval of Memory

For short-term memory retrieval, two-way ANOVA analyses revealed that there was no statistically significant effect caused by oleamide (5 mg/kg) pretreatment [*F*(1.26) = 2.59; *p* = 0.1196], as well as by interactions between oleamide (5 mg/kg) pretreatment and MK-801 (0.3 mg/kg) treatment [*F*(1.26) = 1.23; *p* = 0.2774], but there was a statistically significant effect caused by MK-801 (0.3 mg/kg) treatment [*F*(1.26) = 11.17; *p* = 0.0025]. The post hoc Bonferroni’s test confirmed that MK-801 at the dose of 0.3 mg/kg significantly decreased LI values in mice in the PA test in comparison to the vehicle/vehicle-treated mice, pointing to the amnestic effect of this drug (*p* < 0.01), and that oleamide (5 mg/kg) had no influence on this amnestic effect of MK-801 (0.3 mg/kg) (Fig. 2Ca).

For long-term memory retrieval, two-way ANOVA analyses revealed that there was no statistically significant effect caused by oleamide (5 mg/kg) pretreatment [*F*(1.24) = 1.47; *p* = 0.2377], as well as by interactions between oleamide (5 mg/kg) pretreatment and MK-801 (0.3 mg/kg) treatment [*F*(1.24) = 1.10; *p* = 0.3057], but there was a statistically significant effect caused by MK-801 (0.3 mg/kg) treatment [*F*(1.24) = 25.02; *p* < 0.0001]. The post hoc Bonferroni’s test confirmed that MK-801 at the dose of 0.3 mg/kg significantly decreased LI values in mice in the PA test in comparison to the vehicle/vehicle-treated mice, pointing to the amnestic effect of this drug (*p* < 0.01), and that oleamide (5 mg/kg) had no influence on this amnestic effect of MK-801 (0.3 mg/kg) (Fig. 2Ca).

### The Influence of the Administration of AM 251 on the Memory Impairment Provoked by MK-801 in the PA Test in Mice

#### Acquisition of Memory

**Fig. 3:**
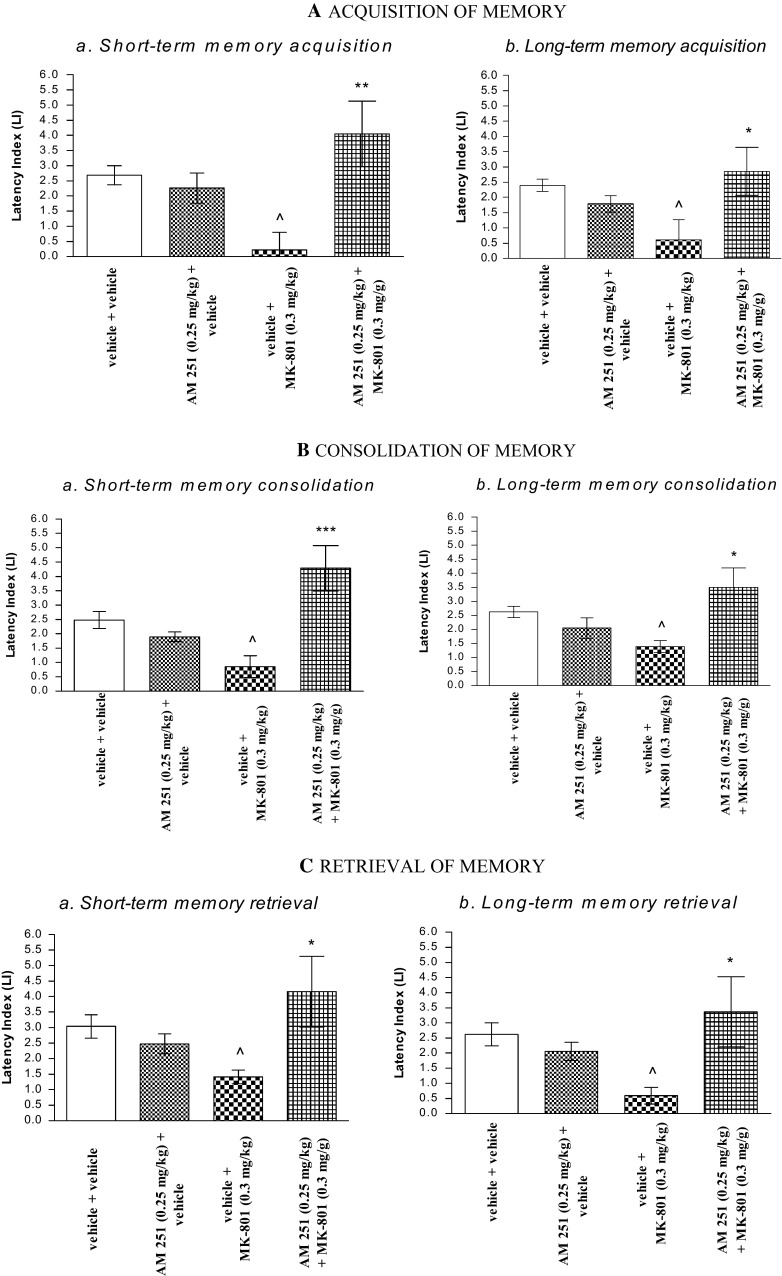
Influence of AM 251 on the memory-related responses, expressed as latency index (LI) during the short-term (**a**) or long-term (**b**) acquisition **(A),** consolidation **(B**), and retrieval **(C)** trial, induced by an acute administration of MK-801, using the PA test in mice. Non-effective dose of AM 251 (0.25 mg/kg, ip) or vehicle was administered 15 min prior to vehicle or effective (0.3 mg/kg, ip) MK-801 injection. All drugs were administered 15 min before the first trial (**A**) or immediately after the first trial (**B**), and mice were re-tested 2 h (for short-term memory) or 24 h (for long-term memory) later. In the case of retrieval of memory (**C**), all drugs were administered 2 h (a) or 24 h (**b**) after the first trial, and mice were re-tested 15 min after the last injection; *n* = 8–12; the mean ± SEM; ^*p* *<* 0.05 vs. vehicle/vehicle-treated group; *p *<* 0.05; ***p *<* 0.01 vs. vehicle/MK-801 (0.3 mg/kg)-treated group; Bonferroni’s test

For short-term memory acquisition, two-way ANOVA analyses revealed that there was no statistically significant effect caused by MK-801 (0.3 mg/kg) treatment [*F*(1.28) = 0.29; *p* = 0.5922], but there was a statistically significant effect caused by AM 251 (0.25 mg/kg) pretreatment [*F*(1.28) = 7.37; *p* = 0.0112] and interactions [*F*(1.28) = 11.52; *p* = 0.0021]. The post hoc Bonferroni’s test revealed that MK-801 at the dose of 0.3 mg/kg significantly decreased LI values in mice in the PA test in comparison to the vehicle/vehicle-treated mice, confirming an amnestic effect of this drug (*p* < 0.05). This amnestic effect of MK-801 (0.3 mg/kg) was reversed by AM 251 (0.25 mg/kg) (*p* < 0.01 vs. vehicle/MK-801(0.3 mg/kg)-treated mice) (Fig. 3Aa).

For long-term memory acquisition, two-way ANOVA analyses revealed that there was a statistically significant effect of interactions between MK-801 (0.3 mg/kg) treatment and AM 251 (0.25 mg/kg) pretreatment [*F*(1.30) = 7.90; *p* = 0.0086], but there was no statistically significant effect of MK-801 (0.3 mg/kg) treatment [*F*(1.30) = 0.51; *p* = 0.4819], as well as AM 251 (0.25 mg/kg) pretreatment [*F*(1.30) = 2.59; *p* = 0.1178]. The post hoc Bonferroni’s test indicated that MK-801 at the dose of 0.3 mg/kg significantly decreased LI values in mice in the PA test in comparison to the vehicle/vehicle-treated mice, confirming an amnestic effect of this drug (*p* < 0.05), and this memory impairment caused by MK-801 (0.3 mg/kg) was attenuated by AM 251 (0.25 mg/kg) (*p* < 0.05 vs. vehicle/MK-801 (0.3 mg/kg)-treated mice) (Fig. 3Ab).

#### Consolidation of Memory

For short-term memory consolidation, two-way ANOVA analyses revealed that there was no statistically significant effect caused by MK-801 (0.3 mg/kg) treatment [*F*(1.29) = 0.78; *p* = 0.3858], but there was a statistically significant effect caused by AM 251 (0.25 mg/kg) pretreatment [*F*(1.29) = 10.74; *p* = 0.0027] and interactions [*F*(1.29) = 21.45; *p* < 0.0001]. The post hoc Bonferroni’s test revealed that MK-801 at the dose of 0.3 mg/kg significantly decreased LI values in mice in the PA test in comparison to the vehicle/vehicle-treated mice, confirming an amnestic effect of this drug (*p* < 0.05). This amnestic effect of MK-801 (0.3 mg/kg) was reversed by AM 251 (0.25 mg/kg) (*p* < 0.001 vs. vehicle/MK-801(0.3 mg/kg)-treated mice) (Fig. 3Ba).

For long-term memory consolidation, two-way ANOVA analyses revealed that there was no statistically significant effect caused by MK-801 (0.3 mg/kg) treatment [*F*(1.25) = 0.06; *p* = 0.802], not quite statistically significant effect caused by AM 251 (0.25 mg/kg) pretreatment [*F*(1.25) = 3.39; *p* = 0.0774], and there was a statistically significant effect caused by interactions between AM 251 (0.25 mg/kg) pretreatment and MK-801 (0.3 mg/kg) treatment [*F*(1.25) = 10.33; *p* = 0.0036]. The post hoc Bonferroni’s test revealed that MK-801 at the dose of 0.3 mg/kg significantly decreased LI values in mice in the PA test in comparison to the vehicle/vehicle-treated mice, confirming an amnestic effect of this drug (*p* < 0.05), and additionally, this amnestic effect of MK-801 (0.3 mg/kg) was reversed by AM 251 (0.25 mg/kg) (*p* < 0.05 vs. vehicle/MK-801(0.3 mg/kg)-treated mice) (Fig. 3Bb).

#### Retrieval of Memory

For short-term memory retrieval, two-way ANOVA analyses revealed that there was no statistically significant effect caused by MK-801 (0.3 mg/kg) treatment [*F*(1.28) = 0.00; *p* = 0.9671], not quite statistically significant effect caused by AM 251 (0.25 mg/kg) pretreatment [*F*(1.28) = 2.92; *p* = 0.0985], and there was a statistically significant effect caused by interactions between AM 251 (0.25 mg/kg) pretreatment and MK-801 (0.3 mg/kg) treatment [*F*(1.28) = 6.74; *p* = 0.0148]. The post hoc Bonferroni’s test revealed that MK-801 at the dose of 0.3 mg/kg significantly decreased LI values in mice in the PA test in comparison to the vehicle/vehicle-treated mice, confirming an amnestic effect of this drug (*p* < 0.05), and additionally, this amnestic effect of MK-801 (0.3 mg/kg) was attenuated by AM 251 (0.25 mg/kg) (*p* < 0.05 vs. vehicle/MK-801(0.3 mg/kg)-treated mice) (Fig. 3Ca).

For long-term memory retrieval, two-way ANOVA analyses revealed that there was no statistically significant effect caused by MK-801 (0.3 mg/kg) treatment [*F*(1.27) = 0.35; *p* = 0.5584], not quite statistically significant effect caused by AM 251 (0.25 mg/kg) pretreatment [*F*(1.27) = 3.34; *p* = 0.0788], and there was a statistically significant effect caused by interactions between AM 251 (0.25 mg/kg) pretreatment and MK-801 (0.3 mg/kg) treatment [*F*(1.27) = 7.60; *p* = 0.0103]. The post hoc Bonferroni’s test revealed that MK-801 at the dose of 0.3 mg/kg significantly decreased LI values in mice in the PA test in comparison to the vehicle/vehicle-treated mice, confirming an amnestic effect of this drug (*p* < 0.05). This memory impairment provoked by MK-801 (0.3 mg/kg) was attenuated by AM 251 (0.25 mg/kg) (*p* < 0.05 vs. vehicle/MK-801(0.3 mg/kg)-treated mice) (Fig. 3Cb).

In the next step, we induced the hyperlocomotion characteristic for schizophrenia (positive symptoms) provoked by the acute administration of MK-801 and evaluated the influence of CB1 receptor ligands on this MK-801-related hyperactivity.

### Hyperactivity of Mice Measured in Actimeters Provoked by an Acute Administration of MK-801

**Fig. 4:**
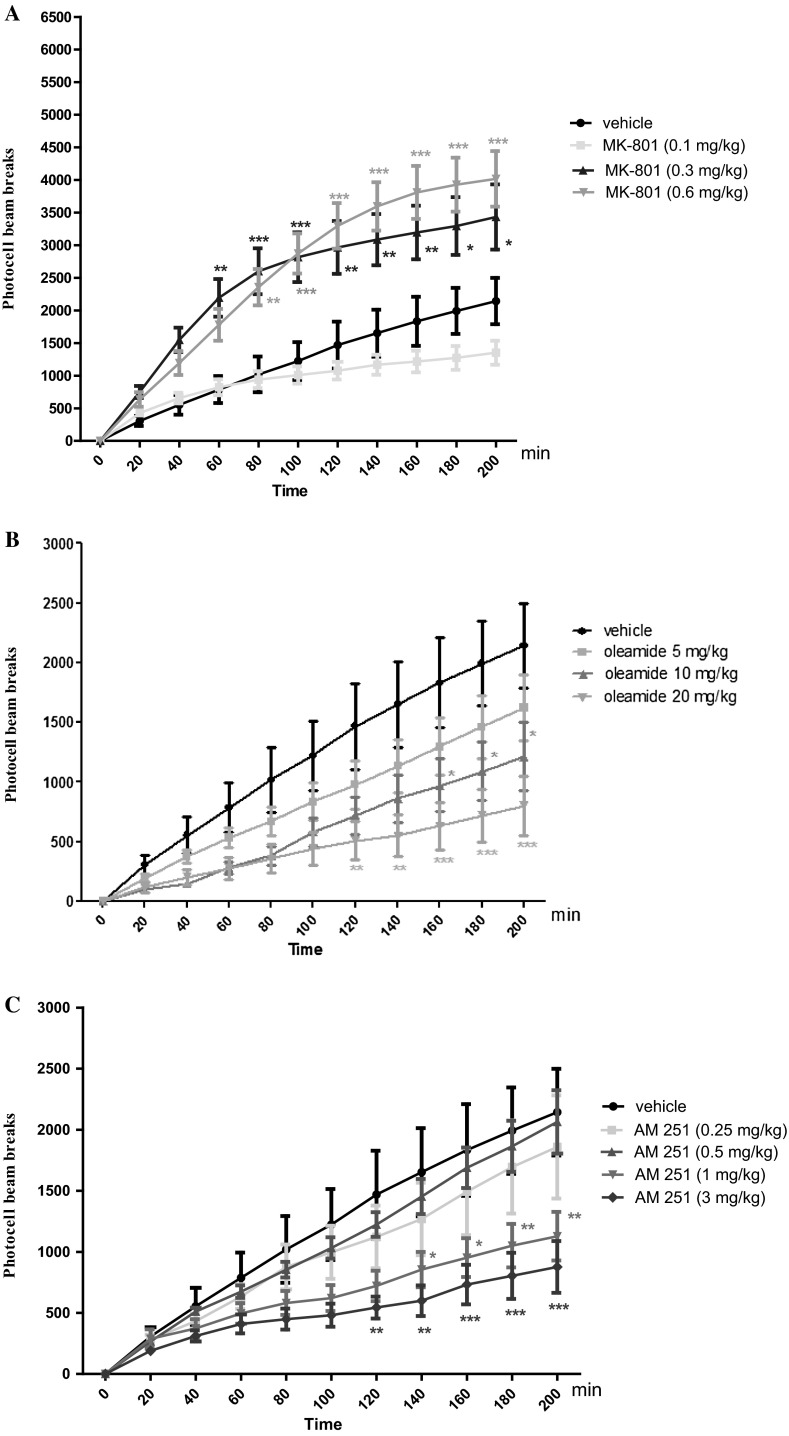
Effects of an acute MK-801 (**A**), oleamide (**B**), AM 251 (**C**) or vehicle administration on the locomotor activity in mice. MK-801 (0.1; 0.3 and 0.6 mg/kg; ip), oleamide (5; 10 and 20 mg/kg; ip), AM 251 (0.25; 0.5; 1 and 3 mg/kg; ip), or vehicle were immediately before the test; *n* = 8–12; the mean ± SEM; *p *<* 0.05; **p *<* 0.01; ***p *<* 0.001 vs. vehicle-treated control group; Bonferroni’s test

Two-way ANOVA analyses revealed that there was statistically significant effect caused by time [*F*(10.264) = 41.83; *p* < 0.0001], and MK-801 (0.1; 0.3 and 0.6 mg/kg) treatment [*F*(3.264) = 91.64; *p* *<* 0.0001], as well as by interactions between time and MK-801 treatment [*F*(30.264) = 2.78; *p* < 0.0001]. The Bonferroni’s test revealed that an acute injection of MK-801 at the dose of 0.3 mg/kg significantly increased locomotor activity of mice between 60 and 200 min of experiment as compared with the vehicle-administered control group (for 60 min of experiments *p* < 0.01; for 80 and 100 min *p* < 0.001; for 120–160 min *p* < 0.01; for 180 and 200 min *p* < 0.05). Similarly, the Bonferroni’s test revealed that an acute injection of MK-801 at the dose of 0.6 mg/kg significantly increased locomotor activity of mice between 80 and 200 min of experiment as compared with the vehicle-administered control group (for 80 min of experiments *p* < 0.01; for 100–200 min *p* < 0.001). MK-801 at the dose of 0.1 mg/kg had no influence on the locomotor activity of mice in comparison to the vehicle-treated control group (Fig. 4A).

### The Influence of CB1 Receptor Agonist, Oleamide, on the Locomotor Activity of Mice

 Two-way ANOVA analyses revealed that there was statistically significant effect caused by time [*F*(10.264) = 22.85; *p* < 0.0001], and oleamide (5; 10 and 20 mg/kg) treatment [*F*(3.264) = 30.26; *p* < 0.0001], but there was no statistically significant effect caused by interactions between time and oleamide treatment [*F*(30.264) = 0.95; *p* = 0.5512]. The Bonferroni’s test revealed that an acute injection of oleamide at the dose of 10 mg/kg significantly decreased locomotion in mice between 160 and 200 min of experiments in comparison to the vehicle-treated control group (*p* < 0.05). Similarly, the Bonferroni’s test revealed that an acute injection of oleamide at the dose of 20 mg/kg significantly decreased locomotor activity of mice between 120 and 200 min of experiment as compared with the vehicle-treated control group (for 120 and 140 min of experiments *p* < 0.01; for 160–200 min *p* < 0.001). Oleamide at the dose of 5 mg/kg had no influence on the locomotor activity of mice in comparison to the vehicle-treated control group (Fig. 4B).

###  The Influence of CB1 Receptor Antagonist, AM 251, on the Locomotor Activity of Mice

Two-way ANOVA analyses revealed that there was statistically significant effect caused by time [*F*(10.330) = 34.81; *p* < 0.0001], and AM 251 (0.25; 0.5; 1 and 3 mg/kg) treatment [*F*(4.330) = 24.32; *p* < 0.0001], but there was no statistically significant effect caused by interactions between time and AM 251 treatment [*F*(40.330) = 1.10; *p* = 0.3203]. The Bonferroni’s test revealed that an acute injection of AM 251 at the dose of 1 mg/kg significantly decreased locomotion in mice between 140 and 200 min of experiments in comparison to the vehicle-treated control group (for 140 and 160 min of experiments *p* < 0.05; 180 and 200 min *p* < 0.01). Similarly, the Bonferroni’s test revealed that an acute injection of AM 251 at the dose of 3 mg/kg significantly decreased locomotor activity of mice between 120 and 200 min of experiment as compared with the vehicle-administered control group (for 120 and 140 min of experiments *p* < 0.01; for 160–200 min *p* < 0.001). AM 251 at the doses of 0.25 and 0.5 mg/kg had no influence on the locomotor activity of mice in comparison to the vehicle-treated control group (Fig. 4C).

Based on the results obtained from these two experiments in the actimeters, the effective doses of MK-801 (0.3 and 0.6 mg/kg) and non-effective doses of oleamide (5 mg/kg) and AM 251 (0.25 and 0.5 mg/kg) were then chosen for the next behavioral experiment evaluating the involvement of CB1 receptors in the MK-801-induced hyperactivity.

### The Influence of the Administration of Oleamide on the Hyperactivity of Mice Provoked by an Acute Administration of MK-801

**Fig. 5:**
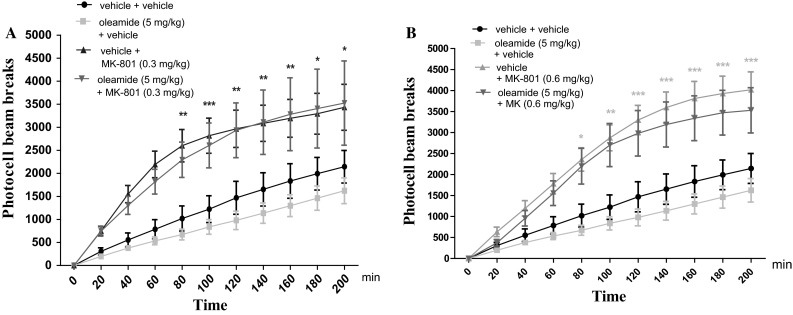
Effect of oleamide on MK-801-induced hyperactivity in mice. Non-effective dose of oleamide (5 mg/kg; ip) or vehicle was administered 15 min prior to vehicle or effective (0.3 mg/kg; ip) **(A)** and (0.6 mg/kg; ip) **(B)** MK-801 injection. After the last injection, the mice were then tested in actimeters; *n* = 8–12; the mean ± SEM; *p *<* 0.05; **p *<* 0.01; ***p *<* 0.001 vs. vehicle/vehicle-treated group; Bonferroni’s test

Two-way ANOVA analyses revealed that there was statistically significant effect caused by time [*F*(10.264) = 22.11; *p* *<* 0.0001], and drugs (MK-801 (0.3 mg/kg) and/or oleamide (5 mg/kg) treatment [*F*(3.264) = 47.13; *p* *<* 0.0001], but there was no statistically significant effect caused by interactions between time and drugs treatment [*F*(30.264) = 0.89; *p* = 0.6358]. The post hoc Bonferroni’s test confirmed that an acute injection of MK-801 at the dose of 0.3 mg/kg significantly increased locomotor activity of mice between 80 and 200 min of experiment as compared with the vehicle/vehicle-injected control group (for 80 min of experiments *p <* 0.01; for 100 min *p <* 0.001; for 120–160 min *p <* 0.01; for 180 and 200 min *p <* 0.05). Oleamide (5 mg/kg) had no influence on MK-801 (0.3 mg/kg)-induced hyperactivity (Fig. 5A). Indeed, two-way ANOVA analyses revealed that there was statistically significant effect caused by time [*F*(10.264) = 39.46; *p* < 0.0001], and drugs (MK-801 (0.6 mg/kg) and/or oleamide (5 mg/kg) treatment [*F*(3.264) = 72.39; *p* < 0.0001], as well as by interactions between time and drugs treatment [*F*(30.264) = 2.06; *p* = 0.0014]. The post hoc Bonferroni’s test revealed that MK-801 at the dose of 0.6 mg/kg significantly increased locomotor activity of mice in actimeters between 80 and 200 min of experiments (for 80 min of experiments *p* < 0.05; for 100 min *p* < 0.01; for 120–200 min *p* < 0.001), in comparison to the vehicle/vehicle-treated mice. Oleamide (5 mg/kg) had no influence on MK-801 (0.6 mg/kg)-induced hyperactivity (Fig. 5B).

### The Influence of the Administration of AM 251 on the Hyperactivity of Mice Provoked by an Acute Administration of MK-801

**Fig. 6:**
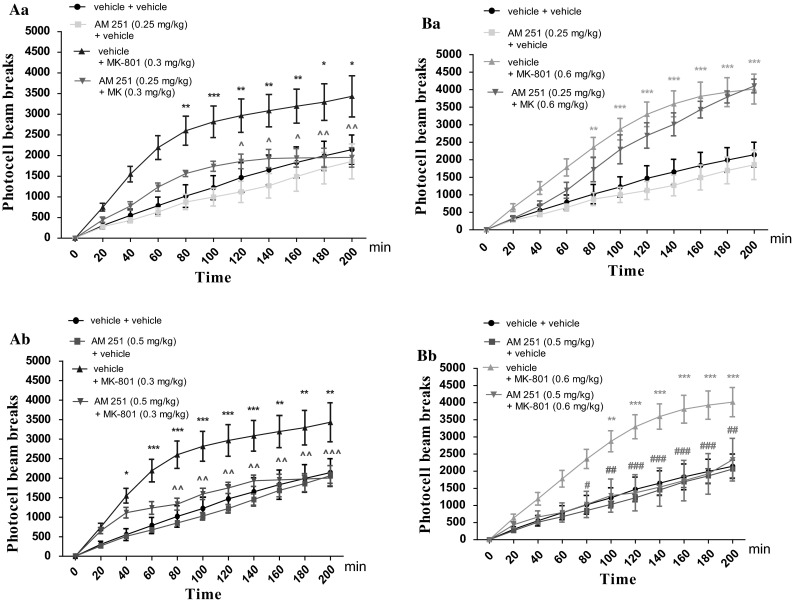
Effect of AM 251 on MK-801-induced hyperactivity in mice. Non-effective dose of AM 251 (0.25 mg/kg; ip) or vehicle was administered 15 min prior to vehicle or effective: (0.3 mg/kg; ip) **(Aa)** and (0.6 mg/kg; ip) **(Ba)** MK-801 injection. Similarly, non-effective dose of AM 251 (0.5 mg/kg; ip) or vehicle were administered 15 min prior to vehicle or effective: (0.3 mg/kg; ip) **(Ab)** and (0.6 mg/kg; ip) **(Bb)** MK-801 injection. After the last injection, the mice were then tested in actimeters; *n* = 8–12; the mean ± SEM; *p *<* 0.05; **p *<* 0.01; ***p *<* 0.001 vs. vehicle/vehicle-treated group; ^*p* *<* 0.05; ^^*p* *<* 0.01; ^^^*p* *<* 0.001 vs. vehicle/MK-801(0.3 mg/kg)-treated mice; ^#^ *p* *<* 0.05; ^##^ *p* *<* 0.01; ^###^ *p* *<* 0.001 vs. vehicle/MK-801(0.6 mg/kg)-treated mice Bonferroni’s test

1. Two-way ANOVA analyses revealed that there was statistically significant effect caused by time [*F*(10.264) = 32.61; *p* < 0.0001], and drugs (MK-801 (0.3 mg/kg) and/or AM 251 (0.25 mg/kg) treatment [*F*(3.264) = 56.55; *p* < 0.0001], but there was no statistically significant effect caused by interactions between time and drugs treatment [*F*(30.264) = 1.12; *p* = 0.3065]. The post hoc Bonferroni’s test confirmed that an acute injection of MK-801 at the dose of 0.3 mg/kg significantly increased locomotor activity of mice between 80 and 200 min of experiment in comparison to the vehicle/vehicle-treated mice (for 80 min of experiments *p* < 0.01; for 100 min *p* < 0.001, for 120–160 min *p* < 0.01, for 180 and 200 min *p* < 0.05). Moreover, this hyperactivity provoked by MK-801 (0.3 mg/kg) was attenuated by AM 251 (0.25 mg/kg) between 120 and 200 min of experiment (for 120–160 min of experiment *p* < 0.05, for 180–200 min *p* < 0.01 vs. vehicle/MK-801 (0.3 mg/kg)-treated mice) (Fig. 6Aa). In turn, for the experiments dealing with MK-801 at the dose of 0.6 mg/kg, two-way ANOVA analyses indicated that there was statistically significant effect caused by time [*F*(10.264) = 55.82; *p* < 0.0001], and drugs (MK-801 (0.6 mg/kg) and/or AM 251 (0.25 mg/kg) treatment [*F*(3.264) = 77.11; *p* < 0.0001], as well as by interactions between time and drugs treatment [*F*(30.264) = 2.79; *p* < 0.0001]. The Bonferroni’s test confirmed that an acute injection of MK-801 at the dose of 0.6 mg/kg significantly increased locomotor activity of mice between 80 and 200 min of experiment as compared with the control vehicle/vehicle-treated mice, (for 80 min of experiments *p* < 0.01; for 100–200 min *p* < 0.001). AM 251 (0.25 mg/kg) had no influence on MK-801 (0.6 mg/kg)-induced hyperactivity (Fig. 6Ba).

2. Two-way ANOVA analyses revealed that there was statistically significant effect caused by time [*F*(10.264) = 40.66; *p* < 0.0001], and drugs (MK-801 (0.3 mg/kg) and/or AM 251 (0.5 mg/kg) treatment [*F*(3.264) = 64.35; *p* < 0.0001], but there was no statistically significant effect caused by interactions between time and drugs treatment [*F*(30.264) = 1.36; *p* = 0.1093]. The post hoc Bonferroni’s test confirmed that an acute injection of MK-801 at the dose of 0.3 mg/kg significantly increased locomotor activity of mice between 40 and 200 min of experiment in comparison to the vehicle/vehicle-treated mice (for 40 min of experiments *p* < 0.05, for 60–140 min *p* < 0.001, for 160–200 min *p* < 0.01). Moreover, this hyperactivity provoked by MK-801 (0.3 mg/kg) was attenuated by AM 251 (0.5 mg/kg) between 80 and 200 min of experiment (for 80–180 min of experiment *p* < 0.01, for 200 min *p* < 0.001 vs. vehicle/MK-801 (0.3 mg/kg)-treated mice) (Fig. 6Ab).

 Additionally, two-way ANOVA analyses revealed that there was statistically significant effect caused by time [*F*(10.264) = 30.66; *p* < 0.0001], and drugs (MK-801 (0.6 mg/kg) and/or AM 251 (0.5 mg/kg) treatment [*F*(3.264) = 52.22; *p* < 0.0001], but there was no statistically significant effect caused by interactions between time and drugs treatment [*F*(30.264) = 1.49; *p* = 0.0549]. The post hoc Bonferroni’s test confirmed that an acute injection of MK-801 at the dose of 0.6 mg/kg significantly increased locomotor activity of mice between 80 and 200 min of experiment in comparison to the vehicle/vehicle-treated mice (for 80 min of experiments *p* < 0.05, for 100 min *p* < 0.01, for 120–200 min *p* < 0.001). Moreover, this hyperactivity provoked by MK-801 (0.6 g/kg) was attenuated by AM 251 (0.5 mg/kg) between 80 and 200 min of experiment (for 80 min of experiment *p* < 0.05; for 100 min *p* < 0.01, for 120–180 min *p* < 0.001, for 200 min *p* < 0.01) vs. vehicle/MK-801 (0.6 mg/kg)-treated mice) (Fig. 6Bb).

## Discussion

The correlation between cannabis and psychosis-like effects has been a matter of debate for a long time. Several lines of experimental and clinical evidence point out at a close relationship between endocannabinoid system and schizophrenia (Kucerova et al. 2014). As we mentioned in Introduction section, CB1 receptor agonists induced memory-related disturbances (Ferrari et al. 1999; Kruk-Slomka and Biala 2016; Kruk-Slomka et al. 2015a; Pamplona and Takahashi 2006), whereas antagonists of this type of receptors facilitated memory and learning processes in rodents evaluated in many memory tasks (Kruk-Slomka and Biala 2016; Kruk-Slomka et al. 2015a; Lichtman 2000; Takahashi et al. 2005; Terranova et al. 1996). The involvement of the CB1 receptors in psychotic-like effects in animal models of schizophrenia has been also reported. CB1 receptor agonists were able to induce effects typical for schizophrenia; in turn, CB1 receptor antagonists had antipsychotic properties observed in rodents (Barzegar et al. 2015; Kucerova et al. 2014; Levin et al. 2012; Roser and Haussleiter 2012). For example, behavioral studies have demonstrated that an acute administration of Δ9-tetrahydrocannabinol (Δ9-THC), the major psychoactive component of cannabis, and a CB1 receptor agonist impaired acquisition of memory evaluated in various models of memory in rodents, e.g., the object recognition task or water maze test (Da and Takahashi 2002; Lichtman et al. 1995). On the other hand, an acute administration of the CB1 antagonist, e.g., rimonabant improved memory processes in the spatial memory test (Robinson et al. 2010). Our previous studies have also confirmed that an acute injection of oleamide, a CB1 receptor agonist, impaired the short-term as well as long-term acquisition, consolidation, and/or retrieval of memory and learning in the IA task. In turn, an acute injection of AM 251, a CB1 receptor antagonist, improved all short-term or long-term memory stages mentioned above. Additionally, this memory impairment induced by oleamide was reversed by AM 251 in mice during the IA test, confirming the influence of CB1 receptors (Kruk-Slomka and Biala 2016).

Based on the data cited above, the aim of the present research was to evaluate the involvement of the endocannabinoid system, through CB1 receptors, in the symptoms typical for schizophrenia in mice, provoked by an acute injection of NMDA receptor antagonist, MK-801, as an animal model of schizophrenia.

Previously, many of biochemical, molecular, and pharmacological studies have demonstrated the functional interactions between CB1 and NMDA receptors (Rodríguez-Muñoz et al. 2012; Sánchez-Blázquez et al. 2014). For example, MK-801 at the dose of 0.1 mg/kg attenuated the analgesic but not the hypothermic responses to Δ9-THC. Indeed, pretreatment with MK-801 strongly reduced the capacity of cannabinoids to produce analgesia (Palazzo et al. 2001). What is more, Barzegar et al. (2015) have shown that AM 251 prevented the somewhat inhibitory effects of MK-801 on acquisition and retrieval in the PA test. However, the close interactions between CB1 and NMDA receptors in the context of schizophrenia-associated behavior have been evaluated in our presented studies for the first time.

Our results are conformable with the psychosis-like effects of MK-801 in animals, observed previously. Chadman et al. (2006) revealed that the systemic administration of MK-801 (0.1 mg/kg) impaired memory and learning processes in rats during phase of retrieval. However, this low dose of MK-801 was not enough to decrease memory acquisition (Ceretta et al. 2008). Similarly to these cited data, our studies confirmed that an acute injection of MK-801 (0.1–0.6 mg/kg) was able to impair variety stages (acquisition, consolidation and retrieval) of short- or/and long memory, as well as was able to induce hyperactivity in mice.

Finally, in the presented studies, we have indicated that an acute injection of CB1 receptor agonist, oleamide (5–20 mg/kg), had no influence on the short- and long-term memory deficits as well as on the hyperlocomotion in mice, provoked by MK-801. The lack of effects of oleamide on the memory impairment or hyperactivity provoked by MK-801 obtained in our experiments may be connected with the fact that oleamide has not been tested yet in details using animal models. Thus, the mechanisms of activity of oleamide remain unknown and are still an area of current research. However, due to the fact that oleamide is structurally related to the endogenous cannabinoid, anandamide, it seems to be able to activate the CB1 receptors as a full agonist, e.g., the memory impairment observed in the IA task (Kruk-Slomka and Biala 2016). However, any effects induced by oleamide may be associated with the interaction not only with these receptors but also with multiple other neurotransmitter systems and receptors. Thus, more detailed knowledge of this CB compound deserves further investigation.

What is of interest, we have also indicated that an amnestic effects or hyperlocomotion induced by MK-801 was attenuated by an acute administration of AM 251 (0.25 and 0.5 mg/kg), a CB1 receptor antagonist.

This strict relationship between endocannabinoid system and schizophrenia-associated effects is connected with many factors, neurotransmitters and receptors. It has been known that endocannabinoid system has a strong impact on the function of many neurotransmitter systems, including those that are involved in the pathophysiology of schizophrenia, e.g., the glutamatergic system. Literature data have shown that endocannabinoid system may have influence especially on the action of NMDA receptor ligands, connecting strictly with psychosis or other schizophrenia-related behavior (Javitt 2007).

It has been revealed that cannabinoids use reduced glutamatergic synaptic transmission in several brain regions involved in the regulation of many memory-related functions (Auclair et al. 2000; Azad et al. 2003; Fujiwara and Egashira 2004; Misner and Sullivan 1999; Robbe et al. 2001). For example, it has been shown that CB1 receptor knockout mice exhibit enhanced LTP of excitatory synaptic transmission (Bohme et al. 2000). What is more, CB1 activation, by the administration of synthetic CB1 receptor agonists, reduced LTP and inhibited release of Glu in the hippocampus (Sullivan 2000). These effects are strongly related to NMDA receptors function, which have been implicated in learning and memory processes (Sánchez-Blázquez et al. 2014).

Several studies have also indicated that cannabinoids have influence on the glutamatergic NMDA-related receptors function through various mechanisms, such as the presynaptic reduction of Glu release into the synaptic cleft (Li et al. 2011) or the inhibition of postsynaptic CB1 receptors, the signaling pathways of which may interfere with those of NMDA receptors (Hampson et al. 2011; Liu et al. 2009; Sánchez-Blázquez et al. 2014). It has been described that the blockade of CB1 receptors by CB1 receptor antagonist, AM 251, produced significant increase in extracellular Glu (Xi et al. 2006). Consistent with this report, the blockade of LTP by CB1 receptor agonists results from a decrease in the probability of Glu release through presynaptic receptors (Hoffman et al. 2007; Misner and Sullivan 1999).

However, it should be noted that other interactions that may occur between the endocannabinoid and glutamatergic systems could be connected with a different mechanism in which the CB1 receptors directly interact with the NMDA receptors to diminish their activity or cannabinoids may reduce Glu release via some other mechanism, not related with CB1 receptors (Sánchez-Blázquez et al. 2014).

In summary, series of biochemical, molecular, pharmacological studies including our presented results have demonstrated functional interactions between the endocannabinoid and glutamatergic systems (Barzegar et al. 2015; Rodríguez-Muñoz et al. 2012; Sanchez-Blazquez et al. 2014). Naturally, the data including those presented in the present manuscript can suggest that CB1 receptor antagonists may have therapeutic properties in schizophrenia or other psychiatric disorders. However, further work is necessary to explain the pharmacological mechanisms on the behavioral level that underlie specific psychosis-related effects induced by CB1 receptor ligands, as well as the mechanism underlying the interactions between CB1 and NMDA receptors.
